# Outcomes of Anterolateral Thigh Flap Reconstruction for Salvage Laryngopharyngectomy for Hypopharyngeal Cancer after Concurrent Chemoradiotherapy

**DOI:** 10.1371/journal.pone.0053985

**Published:** 2013-01-08

**Authors:** Wei F. Chen, Kai-Ping Chang, Chih-Hao Chen, Victor Bong-Hang Shyu, Huang-Kai Kao

**Affiliations:** 1 Division of Plastic and Reconstructive Surgery, Department of Surgery, University of Iowa Hospitals and Clinics, Iowa City, Iowa, United States of America; 2 Department of Otolaryngology – Head and Neck Surgery, Chang Gung Memorial Hospital & Chang Gung University College of Medicine, Taoyuan, Taiwan; 3 Department of Plastic and Reconstructive Surgery, Chang Gung Memorial Hospital & Chang Gung University College of Medicine, Taoyuan, Taiwan; Harvard Medical School, United States of America

## Abstract

**Objective:**

To evaluate the functional and oncological outcomes of anterolateral thigh flap reconstruction for salvage laryngopharyngectomy after concurrent chemoradiotherapy for patients with hypopharyngeal carcinoma.

**Materials/Methods:**

A retrospective review was conducted on patients who underwent pharyngoesophageal reconstruction using anterolateral thigh flap after salvage laryngopharyngectomy for recurrent hypopharyngeal carcinoma between June 2003 and May 2010 at Chang Gung Memorial Hospital. The perioperative morbidity, mortality, functional outcomes, and oncological outcomes were evaluated.

**Results:**

33 patients were entered into the study. The mean follow-up time was 19.5±12.3 months. Recurrent pathological TNM stages included 3 (9.1%), 2 (6.1%), and 28 (84.8%) patients with stage II, III, and IV disease, respectively. Mean ICU stay was 10.3 days and the mean hospital stay was 39.9 days. Peri-operative mortality occurred in one patient (3%). 16 patients (48.5%) developed recipient site complications. Among them, 14 patients (42.4%) developed fistulas and 9 patients (27.3%) developed strictures. Except for 4 patients (12.1%), all achieved varying degree of oral intake with 29 patients (60.6%) being completely independent from tube feeding. The mean interval to start oral intake was 15.1 days. The 5-year overall survival and disease–free survival rates were 51.8% and 53.7%, respectively. The pN status is an independent predictor of overall survival and disease–free survival (*P* = 0.027 and 0.038, respectively).

**Conclusion:**

Pharyngoesophageal reconstruction after salvage laryngopharyngectomy remains challenging even in the experienced hands. Reconstructive microsurgeons who are prepared to take on these cases should be equally well prepared to manage the potential postoperative complications.

## Introduction

Hypopharyngeal cancer has one of the worst prognoses of head and neck cancers because patients are often diagnosed at advanced stages. Chemoradiotherapy (CRT) has emerged as the mainstay of initial treatments of hypopharyngeal squamous cell carcinoma. In comparison to primary laryngopharyngectomy, it promises organ preservation and provides comparable survival benefits. Local recurrence after CCRT, however, remains a challenging problem. Salvage laryngopharyngectomy is considered to be the most effective treatment, especially in long-term disease control and restoration of oral intake. However, the technical difficulty of both tumor ablation and reconstruction, the expectedly high perioperative morbidity and mortality rates, the diminished quality of life due to functional sacrifice, and the overall poor prognoses of the patients with disease recurrence make the choice of salvage surgery for treatment of locally recurrent hypopharygeal cancer controversial.

Accepted reconstructive modalities include anterolateral thigh (ALT) flap, radial forearm flaps, jejunal flap, gastro-omental flap, and pectoralis flap. The choice of flap depends on individual surgeon’s expertise and preference. A consensus on the flap selection has not been reached [1]–[4].

Our favorable and rather extensive experience of ALT flap in head and neck reconstruction had led us to use this versatile flap as the flap of choice in pharyngoesophageal reconstruction after salvage laryngopharyngectomy [5]. The advantages of ALT flap include consistent and reliable anatomy, long vascular pedicle, being far from the ablative site and allowing a two-team approach, the feasibility to create multiple skin paddles by recruiting additional perforators, the flexibility to reconstruct composite defects by recruiting different tissue types (adipose, muscle, and fascial components) all based on a single pedicle, and low donor site morbidity [6].

The aim of this study was to review our experience of using ALT flap for pharyngoesophageal reconstruction in patients undergoing salvage laryngopharyngectomy for disease recurrence after CCRT. We analyzed the oncological outcomes, complication rates, and the postoperative functional status of these patients.

## Materials and Methods

### Patients

This study was approved by the ethics committee of Chang Gung Memorial Hospital – Linkou Medical Center. Informed consent was not needed since the data were analyzed anonymously. The ethics committee specifically waived the need for consent. A retrospective review was conducted on all patients who underwent pharyngoesophageal reconstruction after salvage laryngopharyngectomy for recurrent hypopharyngeal carcinoma between June 2003 and May 2010 at Chang Gung Memorial Hospital (CCMH), Taiwan. Only patients who had resectable tumors and received ALT flap reconstruction when local recurrence was detected after CCRT were included in the study. All patients were regularly followed every two months until either October 2011 or death.

### Reconstruction

**Figure 1:**
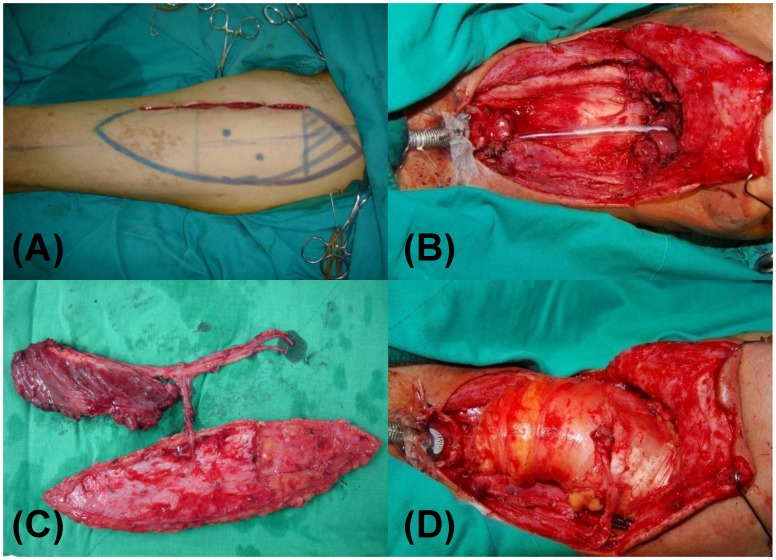
Pharyngoesophageal reconstruction with the anterolateral thigh flap after total laryngopharyngectomy. (A) Preoperative marking. (B) After salvage pharyngolaryngectomy with neck dissection, an “empty” neck was noted. (C) A chimeric flap was composed of a skin paddle and a piece of vastus lateralis (VL) muscle. The skin paddle was tubularized to form a neoesophagus and the VL muscle was used to increase tissue bulk and to obliterate the dead space. (D) Immediate photograph after reconstruction.

The pharyngoesophageal reconstruction was circumferential in 30 patients (90.9%) and near-circumferential in 3 patients (9.1%). All were reconstructed with ALT flaps. The design and harvest of the ALT flaps were performed as previously described [5], [6]. When additional bulk was needed, vastus lateralis (VL) muscle was recruited in a chimeric fashion to provide the needed volume (Fig. 1). 16 patients (48.5%) received ALT fasciocutaneous flap and 17 patients (51.5%) received ALT-VL chimeric flap. The ALT flap was tubularized either by itself or in combination with residual pharyngeal mucosa to form a neoesophagus. The average length of the flap was 16.2±5.8 cm (range, 8–25 cm) and the average width of the flap was 8.6±1.6 cm (range, 6–12 cm). When anterior neck resurfacing was needed and given permissible anatomy, a separate skin island based on independent perforator was harvested for this purpose. Contralateral superior thyroid artery (STA) was preferentially used as recipient artery as it tended to be minimally affected by CCRT. Contralateral transverse cervical artery was used as an alternative when STA was not available.

### Postoperative Care

All patients were admitted to a specialized microsurgery intensive care unit (ICU) postoperatively for flap monitoring. Tube feeding was started on the first postoperative day. The patients were routinely transferred to regular wards on postoperative day 8. Liquid diet was started as soon as a contrast esophagogram on postoperative day 10 demonstrated intact anastomoses. The diet was advanced as tolerated. Fistulae identified on the contrast esophagram were managed conservatively. Surgical treatments were performed when the conservative management failed.

### Statistical Analysis

All data is described as mean ± SD. Statistical analyses was performed using SAS software (version 9.1, SAS institute Inc., Cary, NC). Survival analysis was plotted using the Kaplan-Meyer method and differences were evaluated using the log-rank test. Univariate and multivariate regression analyses were used to define specific risk factors for overall survival and disease-free survival. All *P* values were 2-sided and statistical significance was accepted when *P*<0.05.

## Results

### Patient Characteristics

During the study period, 47 patients underwent pharyngoesophageal reconstruction for salvage ablation for local recurrence. After excluding those reconstructed with non-ALT flaps, a cohort of 33 patients remained. The mean follow-up period was 2.6 years (range, 0.6–6.9 years). All patients were male, with a mean age of 57.4 years (Table 1). Mean body mass index (BMI) was 21.8 (range, 16.5 to 28.6).

**Table 1:** Patients’ details.

	No. of patients (%)
**Age (yrs)**	
Range	39–79
Mean (SD)	57.4 (9.4)
**Gender**	
M	33 (100)
**BMI** a	
Range	28.6–16.5
Mean (SD)	21.8 (3.3)
≦20	13 (39.4)
20–25	13 (39.4)
≧25	7 (21.2)
**Location**	
Piriform sinus	29 (87.8)
Postcricoid	2 (6.1)
Post-pharyngeal wall	2 (6.1)
**T stage**	
T2	4 (12.1)
T3	1 (3.0)
T4	27 (81.8)
**N stage**	
N0	20 (60.6)
N1	5 (15.2)
N2	8 (24.2)
**Overall Stage**	
II	3 (9.1)
III	2 (6.1)
IV	28 (84.8)

a body mass index.

### Operative Details

Intraoperative data are summarized in Table 2. The operative time was 730.7±123.5 min (range, 436–790 min). This represented the combined operative time of the ablative and reconstructive procedures, as a two-team approach was consistently used. The intraoperative blood loss was 312.1±207.7 mL (range, 50–1100 mL). Primary closure of the donor site was achieved in 28 patients (84.8%) of the patients.

**Table 2:** Operative data.

	No. of patients (%)
**Operative time (min)**	
Range	436–790
Mean (SD)	730.7 (123.5)
**Blood loss (mL)**	
Range	50–1100
Mean (SD)	312.1 (207.7)
**ICU** f **stay (day)**	
Range	6–41
Mean (SD)	10.3 (8.3)
**Hospital stay (day)**	
Range	13–92
Mean (SD)	39.9 (22.1)
**In-hospital death**	1 (3.0)
**Flap related**	
** Defect**	
Circumferential	30 (90.9)
Near-Circumferential	3 (9.1)
** Type**	
ALT^a^ skin tube	16 (48.5)
ALT+chimeric VL^b^ muscle	17 (51.5)
** Flap width** **(cm)**	
Range	6–12
Mean (SD)	8.6 (1.6)
** Flap length** **(cm)**	
Range	8–25
Mean (SD)	16.2 (5.8)
** Recipient artery**	
STA^c^	29 (87.9)
TCA^d^	4 (12.1)
** Donor site closure**	
Primary	28 (84.8)
STSG^e^	5 (15.2)

a anterolateral thigh;

b vastus lateralis;

c superior thyroid artery;

d transverse cervical artery;

e split thickness skin graft;

f intensive care unit.

### Postoperative Course and Morbidities

Mean ICU stay was 10.3 days (range, 6–41 days) and the mean hospital stay was 39.9 days (range, 13–92 days) (Table 2). There was 1 (3.0%) in-hospital mortality. Recipient site complications developed in 16 patients (48.5%) and 11 patients (33%) experienced more than one complication (Table 3). Reoperation within 7 days from the initial surgery was necessary in 5 patients (15.2%). Among patients who underwent reoperative surgery, one had unsalvageable flap failure and was replaced with pectoralis major flap, two had venous insufficiency that were successfully salvaged, and the remaining two developed carotid blowout manifested as acute neck hematoma. Both cases of carotid blowout were successfully treated with vessel ligation without neurologic sequelae.

**Table 3:** Postoperative complications.

	No. of patients (%)
**Recipient site**	
Partial necrosis	4 (12.1)
Flap failure	1 (3.0)
Venous insufficiency	2 (6.1)
Neck infection	15 (45.5)
Hematoma	2 (6.1)
Carotid artery blow out	2 (6.1)
Re-operation within 1 week	5 (15.2)
Fistula	14 (42.4)
Stricture	9 (27.3)
**Donor site**	
Seroma	1 (3.0)
Hematoma	1 (3.0)
Infection	1 (3.0)
Wound dehiscence	1 (3.0)
**Medical complications**	
Cardiac	1 (3.0)
Pulmonary	2 (6.1)
Renal	1 (3.0)
Hepatic	1 (3.0)

Esophagocutaneous fistulae were identified in 14 patients (42.4%) upon barium contrast study. Among them, 5 fistulae responded favorably to conservative management and went on to complete healing without surgical intervention. In the remaining 9 cases that failed conservative management, 5 underwent successful surgical revision; the remaining 4 patients could not undergo revision due to disease recurrence.

During follow-up, 9 patients (27.3%) developed anastomotic strictures, 7.2±5.1 months (range, 3–12 months) after surgery. 2 patients developed strictures at upper anastomosis (pharyngeal junction) and 7 patients developed strictures at the lower anastomosis (thoracic esophageal junction). All were successfully treated by mechanical dilatation and the average duration of treatment was 3.3 months.

### Swallowing Function

The time interval from the time of operation to the time of oral intake was 15.1±6.2 days (range, 9–30 days) (Table 4). Swallowing function was determined by the highest level of diet achieved after surgery. Among them, 29 patients (87.9%) achieved oral intake. 23 patients (69.7%) achieved soft or liquid diet. 20 patients (60.6%) were completely independent from tube feeding. 6 patients remained partially dependent on tube feedings due to strictures. Four patients remained completely dependent on tube-feeding due to persistent fistulas.

**Table 4:** Postoperative diet.

Interval to start oral intake, d, mean±SD(range)^a^	15.1±6.2 (9–30)
**Diet, no. (%)**	
Soft	20 (60.6)
Liquid	3 (9.1)
Partial TF^b^	6 (18.2)
Total TF	4 (12.1)

a 29 patients achieve oral intake.

b tube feeding.

### Pathology

All recurrent disease was local, regional, or both. None of the patients had distant metastasis after preoperative systemic work-ups. Recurrent pathological T stage included 4 (9.1%), 1 (6.1%), and 28 (84.8%) patients with T2, T3, and T4, respectively. 13 patients (39.4%) had lymph node metastasis, in which 5 patients (15.2%) had N1 and 8 patients (24.2%) had N2 (Table 1). The overall stages included 3 (9.1%), 2 (6.1%), and 28 (84.8%) patients with stage II, III, and IV disease, respectively. After ablative resection, the pathological examination demonstrated the nearest resection margin to be 4±2.9 mm (range, 0–20 mm).

### Disease Control and Survival

**Figure 2:**
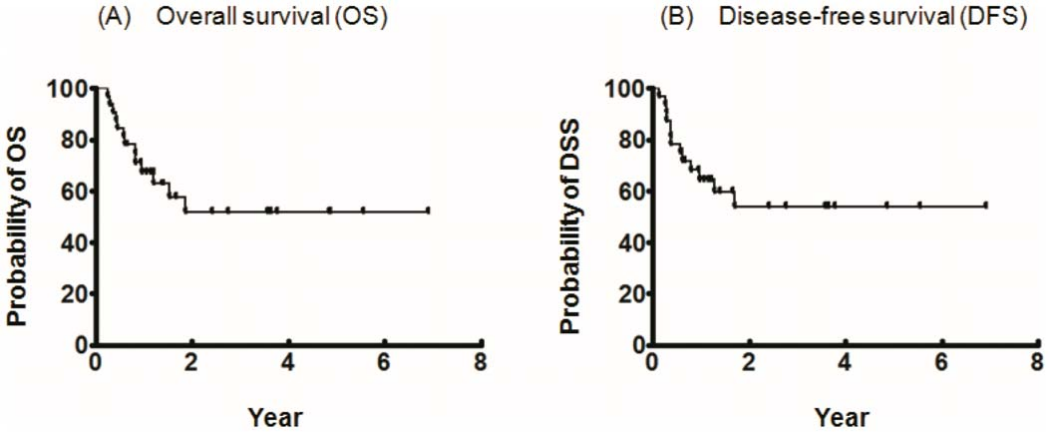
Kaplan-Meier survival analysis in patients with recurrent hypopharyngeal cancers after concurrent chemoradiotherapy receiving ablating salvage surgery and anterolateral thigh flap reconstruction. (A) & (B), Kaplan-Meier plots indicated that 5-year overall survival (OS) and disease-free survival (DFS) were 51.8% and 53.7%, respectively.

The 5-year overall survival and disease-free survival for this specific group of patients were 51.8% and 53.7%, respectively (Figure 2).

### Correlation of BMI, Surgical Margins and Nodal Status (pN) to Patient Overall Survival (OS)

**Figure 3:**
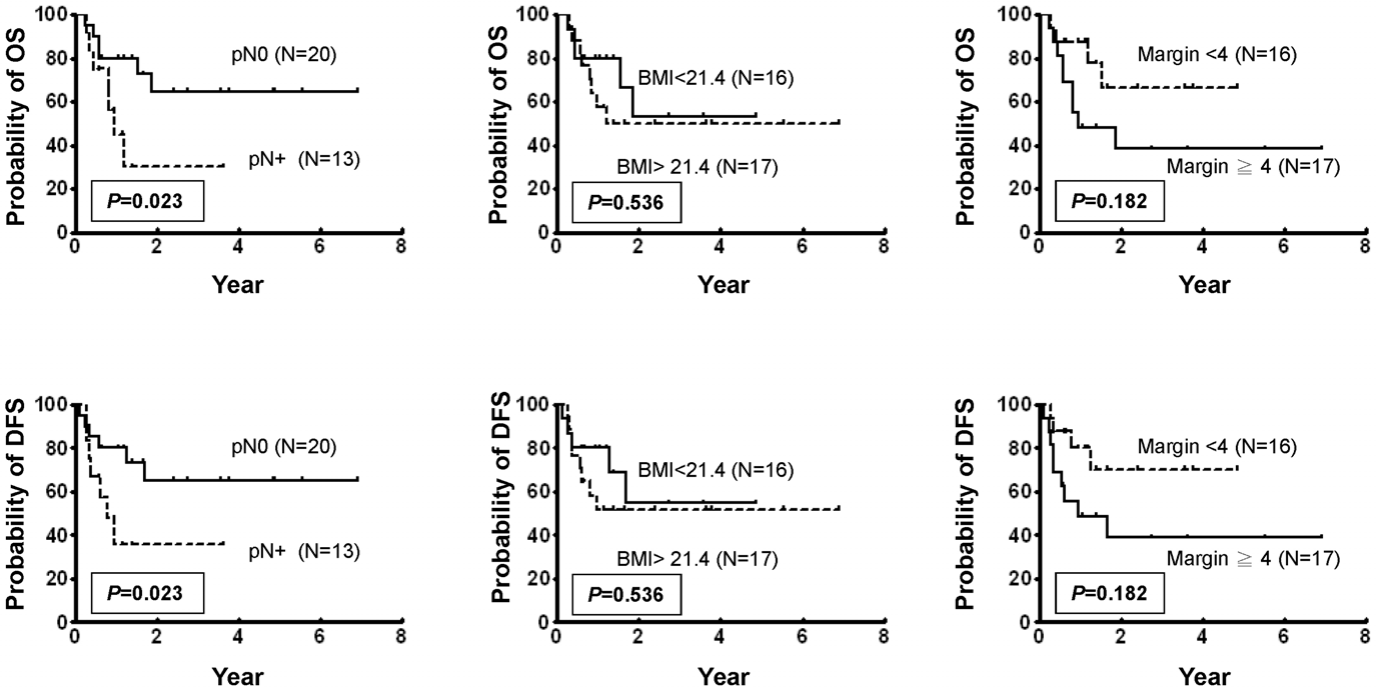
Kaplan-Meier plot analyses of the overall survival (OS) and disease-free survival (DFS) of patient subgroups stratified by pathological nodal status (pN), BMI values, and surgical margins (mm). The inlets indicate statistically significant differences measured by log-rank test.

Using data from these 33 patients undergoing salvage resection and free ALT flap reconstruction, we further evaluated whether BMI, surgical margins, and pN status had any impact upon OS. BMI and surgical margins were stratified into two groups by median, and pN status was stratified by pN0 and pN+. We found that the 3-year survival of pN0 and pN+ was 65.1% and 32.9%, respectively. This difference in OS was significant when compared using a log-rank test (*P* = 0.034) (Figure 3). Conversely, 3-year OS shown by Kaplan-Meier plots of patients stratified by lower and higher BMI values was not significantly different (50.0% and 50.1%, respectively; *P* = 0.749) (Figure 3). Likewise 3-year OS of the patients stratified by lower and higher surgical margins were not significantly different either (38.5% and 62.7%, respectively; *P* = 0.182). To determine whether pN status is an independent predictor of OS, a multivariate analysis was carried out using age, gender, pT status, pN status, BMI values and surgical margins as parameters. We found pN status was the only independent predictors of OS (*P* = 0.027).

### Correlation of BMI, Surgical Margins and pN Status to Patient Disease-free Survival (DFS)

We also evaluated whether BMI, surgical margins, and pN status have any impact on DFS and found that the 3-year survival of pN0 and pN+ were 65.1% and 32.9%, respectively. This difference in DFS was significant when compared using a log-rank test (*P* = 0.034) (Figure 3). Conversely, 3-year DFS shown by Kaplan-Meier plots of patients stratified by lower and higher BMI values was not significantly different (51.4% and 51.7%, respectively; *P* = 0.711) (Figure 3). Likewise 3-year DFS of the patients stratified by lower and higher surgical margins were not significantly different either (38.8% and 66.0%, respectively; *P* = 0.149). To determine whether pN status is an independent predictor of DFS, a multivariate analysis was carried out using age, gender, pT status, pN status, BMI values and surgical margins as parameters. The results indicate that only pN status is an independent predictor of DFS (*P* = 0.038), and the remaining factors are not. Overall, these results indicate that only pN status has a vital impact on OS in these patients through survival analyses.

## Discussion

CCRT as initial treatment for hypopharyngeal carcinoma has recently gained popularity due to its comparable disease control and the potential for organ preservation when compared with primary surgery [7]–[12]. In accordance with the literature, our experience at CGMH demonstrated no survival difference between patients receiving primary CCRT and those receiving primary radical surgery [7], [8]. However, when those who received primary CCRT develop recurrent disease and now require salvage ablation, the reconstruction becomes more challenging than ever. More so than reconstruction after primary laryngopharyngectomy, these patients are compromised by poor nutrition, cachexia, depression, and other morbidities related to chemotherapy and radiation.

Over the past 10 years, we have consolidated our approach for pharyngoesophageal reconstruction. We have increasingly relied on ALT for parital and circumferential reconstructions and jejunum flaps for circumferential reconstructions [5], [13]. For the patients in this study, all of whom had severely compromised physiologic reserves, we favored the ALT flap over the jejunum flap to avoid the morbidities associated with open abdominal surgery, such as postoperative ileus, risks of bowel adhesion and obstruction, incisional hernia, and pulmonary embarrassment. ALT flap also excels over jejunum in producing superior tracheoesophageal speech while providing similar fistula and stricture rates, as demonstrated by Yu et al [14]. The thickness of an ALT flap could be problematic in obese patients. However, this had not been an issue in our study population. None of the patients, even those who were previously obese, had thick thigh tissue when presented for salvage ablation and reconstruction. This was not surprising considering their disease courses and the treatment histories. Before salvage surgery, almost all of the patients could not tolerate oral intake and experienced malnutrition. The skin laxity secondary to soft tissue wasting ironically facilitated ALT donor site closure. Primary closure was achieved in 84.8% of patients despite the harvests of relatively large flaps.

The postoperative complication rate was an important parameter for surgical success because it had significant impact on the hospitalization length, permanent sequelae, functional status, and quality of life. Pharyngocutaneous fistula is the most common early complication after major laryngopharyngeal surgery, reported from 5% to 65% [15]. Our rate of fistula of 42.4% was high when compared with other studies of pharyngoesophageal reconstruction using ALT flap, which reported fistula rates ranging from 0 to 25 percent [2]–[4], [16]. This was likely secondary to our selection criteria of including only the patients with disease recurrence after CCRT. With preoperative CCRT, cancer recurrence, aggressive salvage surgery, and significant co-morbidities, the tissue’s capacity to heal was likely compromised. Tsou et al. found preoperative CCRT to be an independent risk factor that significantly raised the risk of fistula formation, from 21.4% in 112 patients receiving primary total laryngopharyngectomy and reconstruction to 58.3% in 48 patients undergoing post-irradiated salvage laryngopharyngectomy and reconstruction [17].

Compared to previously published literature, in which 5 to 25 percent of transferred flaps require re-exploration within the first week postoperatively due to circulatory compromise or neck hematoma, the re-operative rate of 15.2% in the present study is concerning, especially considering that 2 patients (6.1%) developed life-threatening vascular blowouts [18], [19]. This relatively high re-operative rate, again, reflects the highly morbid nature of the patient population. At the same time, it emphasizes the importance of ICU monitoring. We routinely monitor these patients for a week in a specialized microsurgical ICU. The successful treatment of vascular blowouts and flap salvage was attributed to timely diagnosis and prompt return to the operating room.

To reduce these complications, the ALT flap can be adjusted with the inclusion of partial VL muscle to meet the requirements of the reconstruction, such as increasing tissue bulk, obliteration of dead space, or coverage of the great vessels in the neck. Moreover, the underlying fascia of the ALT flap could provide a vascularized second layer to reinforce the suture lines and mucosal anastomoses.

We found donor wound healing to be reflective of patients’ tissue healing capacity, and useful as an easily monitored tool in assessing patients’ overall metabolic status. Delayed donor wound healing indicates an underlying cause that tipped the balance toward catabolic metabolism. The cause was most frequently poor nutrition in the study’s patient population.

Variable results of postoperative locoregional control and survival have been reported. Kadota et al. reported the results of an analysis on 14 patients who underwent salvage surgery after definitive chemoradiotherapy in a single-institution study. The 5-year disease-free survival and local control rates were 57.1% and 92.9%, respectively [20]. However, less favorable results have also been reported. In a series reported by Relic et al., only 2 out of 20 patients undergoing surgery for histologically proven recurrence after radiochemotherapy (10%) were actually tumor-free and alive after a mean follow-up period of 43.9 months [21]. In our study, the 5-year overall survival and disease-free survival rate for this specific group of patients were 51.8% and 53.7%, respectively. Our results also indicate that only pN status has a vital impact on the overall survival rates. It should be noted that all of the patients in our study had favorable response to CCRT initially and only those with feasibility of complete surgical resection were included. This may introduce selection bias and mask the negative oncological outcomes in the study.

### Conclusion

Despite advances in microsurgical techniques, pharyngoesophageal reconstruction after salvage laryngopharyngectomy remains challenging. Meaningful oncologic and functional outcomes can be obtained by meticulous attention to surgical techniques and postoperative care. Among the techniques available, we continue to favor the ALT flap as it proves to be reliable and versatile. Reconstructive microsurgeons who are prepared to take on these cases should be equally well prepared to manage the potential postoperative complications.
